# Real-world effectiveness of antibiotics in addition to oral corticosteroids for managing asthma exacerbations in adults

**DOI:** 10.1136/bmjresp-2025-003506

**Published:** 2025-10-20

**Authors:** Irene Mommers, Sumaira Mubarik, Job F M van Boven, Jens H Bos, Maarten J Bijlsma, Eelko Hak

**Affiliations:** 1 Groningen Research Institute of Pharmacy, Department of PharmacoTherapy, Epidemiology & Economics, University of Groningen, Groningen, Netherlands; 2 Department of Clinical Pharmacy & Pharmacology, University Medical Centre Groningen, Groningen, Netherlands; 3 Groningen Research Institute for Asthma and COPD (GRIAC), University Medical Centre Groningen, Groningen, Netherlands

**Keywords:** Asthma, Asthma Pharmacology, Asthma Epidemiology, Asthma in primary care, Clinical Epidemiology, Inhaler devices

## Abstract

**Background:**

Antibiotics are widely used to manage acute asthma exacerbations, despite little evidence for their effectiveness. This study assesses the added value of antibiotics alongside oral corticosteroids (OCSs) in treating asthma exacerbations.

**Methods:**

This retrospective cohort study included individuals from the Netherlands between 1994 and 2022 from the IADB.nl pharmacy dispensing database. Individuals had to be 16–45 years old, use inhaled asthma medication and have a first recorded prednisone/prednisolone (OCS) dispense of ≥30 mg/day for 3–14 days. Patients were compared regarding treatment failure (a new dispense of OCS or antibiotics, 15–30 days after initial dispense), based on whether or not they were dispensed antibiotics (AB) alongside their first recorded OCS dispense. Regression analyses with inverse probability of treatment weighting were used to adjust for various confounders.

**Results:**

Of the 5401 individuals included, 38% received antibiotics alongside the first-recorded OCS dispense, with a decreasing trend from 47% in 2009 to 24% in 2020. The OR for treatment failure was 1.36 (95% CI 0.81 to 2.16) for AB+OCS versus OCS-only. The HR for a new exacerbation within 31–365 days of follow-up was 1.20 (95% CI 0.92 to 1.56) for AB+OCS versus OCS-only. The lack of beneficial effect of AB was consistent across subcohorts.

**Conclusions:**

This study found no reduction in treatment failure, nor in risk of subsequent exacerbation, from adding AB to OCS for treating acute asthma exacerbations. We suggest that antibiotics should not be used in primary care settings to treat acute asthma exacerbation unless there are clear signs of bacterial infection.

**WHAT IS ALREADY KNOWN ON THIS TOPIC**

- Despite guideline recommendations against it, antibiotics are widely used alongside oral corticosteroids to manage acute asthma exacerbations in clinical practice. Previous research reported inconsistent results and has not reached a definitive conclusion yet.

**WHAT THIS STUDY ADDS**

- This study reports the extent to which add-on antibiotics are used for the treatment of acute asthma exacerbations in the Netherlands and demonstrates that add-on antibiotics provide no beneficial effect regarding the risk of treatment failure or subsequent exacerbation in primary care settings.

**HOW THIS STUDY MIGHT AFFECT RESEARCH, PRACTICE OR POLICY**

- This research highlights the need to reduce the unjustified prescription of antibiotics in the management of acute asthma exacerbations without clear signs of bacterial infection.

## Introduction

 Asthma is a chronic disease characterised by inflammation of the lower airways, with a prevalence of over 262 million worldwide.^1^ It expresses itself by recurring exacerbations, with symptoms such as bronchospasms, dyspnoea, mucus hypersecretion, cough, wheezing and tightness of the chest.^2 3^ Asthma exacerbations can be triggered by various external factors, for example, viral infections, allergens, air pollution, seasonal changes or poor treatment adherence.^4^

Exacerbations of asthma are typically managed with oral corticosteroids (OCS) (prednisone/prednisolone), and sometimes, antibiotics are indicated. However, the Global Initiative for Asthma (GINA) 2024 report advises against routine antibiotic use, unless there is strong evidence of bacterial infection.^4^ Nevertheless, antibiotic prescriptions for exacerbations in both children and adults remain widespread in clinical practice. The proportion of emergency department (ED) visits for asthma that resulted in AB prescription ranges from 16% to 22% or even higher, while bacterial infections are only found in 7% of acute exacerbations among adults.^5^,^10^ These circumstances differ substantially from chronic obstructive pulmonary disease (COPD). With up to half of COPD exacerbations attributed to bacterial infections, antibiotics have a more prominent role in the treatment of COPD exacerbations.^11^,^13^

A 2018 Cochrane review investigating the efficacy of antibiotics in the treatment of asthma exacerbations found inconsistent outcomes across eight randomised clinical trials.^14^ With mostly in-hospital settings, exclusion of suspected bacterial infections, and possible publication bias, the generalisability of the included studies was limited, and the authors stated to have low confidence in the effect estimates.^14^ In addition, a cohort study investigating the association of antibiotic treatment with outcomes in patients hospitalised for asthma exacerbations and treated with systemic corticosteroids found a small increase in hospital stay duration and higher hospitalisation costs, suggesting even a potentially harmful effect.^15 16^ Lastly, a retrospective cohort study into the effectiveness of antibiotics next to OCS in the management of exacerbations found a slightly decreased risk of having an asthma or wheeze consultation within 2 weeks of follow-up after penicillin, but not macrolides, and an increase in exacerbations among adults within 6 weeks of follow-up.^10^ Yet none of these real-world studies investigated the subsequent exacerbations requiring OCS beyond 12 weeks in primary care settings.

There remains a need for real-world studies to gain insight into the short- and long-term effectiveness of add-on antibiotics to guide their appropriate use,^17^ as existing evidence for add-on antibiotics in managing acute asthma exacerbations is contradictory. Its indications and benefits remain poorly documented,^5^ particularly in primary care settings. Therefore, this study aims to evaluate the added value of antibiotic treatment alongside OCSs for managing acute asthma exacerbations among adults.

## Methods

### Study design

**Figure 1:**
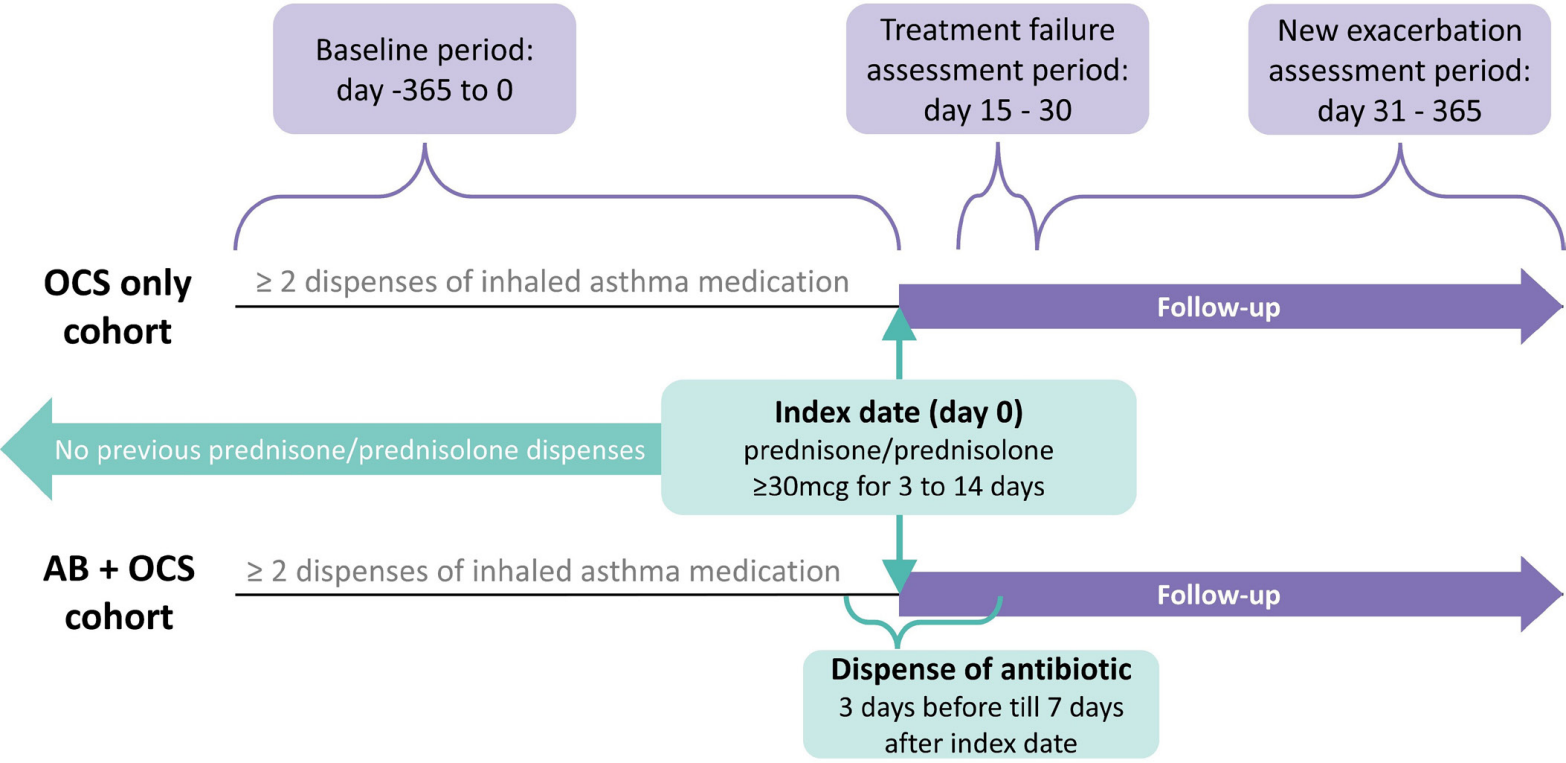
Diagram illustrating the study design. AB, antibiotic; OCS, oral corticosteroid.

We conducted a retrospective, observational cohort study among young adults with exacerbating asthma, comparing those who filled their first short-course of high dose prescription of prednisolone or prednisone (Anatomical Therapeutic Chemical (ATC) codes H02AB06 or H02AB07, ≥30 mg/day for 3–14 days) without antibiotics (OCS-only cohort) to those who were first dispensed prednisone/prednisolone together with antibiotics (AB+OCS cohort) (figure 1).

This study was reported according to the reporting of studies conducted using observational routinely collected health data statement for pharmacoepidemiology (RECORD-PE).^18^

### Study setting and data source

We used data from 1994 to 2022 from the University of Groningen IADB.nl database, which contains dispensing data from over 125 community pharmacies in the North of the Netherlands,^19^ and covers up to 1.3 million patients. Prescription dispensing records contain anonymised patient identifiers and information on the date of dispensing, quantity dispensed, prescribed dosing regimen, number of days for which the prescription was valid, the prescribing physician and the corresponding ATC classification code. Demographic variables available for each patient included date of birth and sex. Clinical data such as biomarkers or physical lung measurements were not available. The dispensing records for each individual are virtually complete, except for medication dispensed during hospitalisation. The dispensing rates from the IADB.nl database have been found representative of the entire Dutch population.^20^

### Study population

To enter the study, individuals aged 16–45 years needed to have a first recorded dispensed prednisone or prednisolone (=index date) of ≥30 mg/dose for 3–14 days and a follow-up period of 365 days. They also need to be present in the dataset one year before the index date, during which they need to have filled at least two prescriptions of the same short- or long-acting selective β2-adrenoreceptor agonist (SABA or LABA), inhaled corticosteroid (ICS) or a combination thereof. To reduce the risk of including patients who may use OCS for other indications than asthma, we excluded individuals who were dispensed antineoplastic agents, antivirals or immunosuppressants (ATC-codes starting with L01, J05 or L04) during the year before index date. To minimise the risk of including patients with COPD, individuals who were ever dispensed roflumilast (ATC-code R03DX07) or who have used LABA without ICS were excluded. Missing dosage information, either for OCS dispensed at index date or for all dispenses of ICS, also resulted in exclusion.

We excluded patients over 45 years old to minimise misclassification, as the medications from the inclusion criteria can also be prescribed for COPD (including emphysema and bronchitis) or other lung conditions, such as lung cancer or fibrosis. Patients with self-reported cancer were excluded from this study. Other lung conditions (eg, idiopathic, pulmonary or cystic fibrosis) have low prevalences (<1 per 1000) as compared with asthma (about 1 in 12 adults).^21^,^23^ COPD incidence increases with age and peaks in late adulthood, while asthma incidence peaks in childhood.^24^ By restricting our study population to young adults, we ensure that the proportion of included patients with lung conditions other than asthma is low.

### Exposure and outcomes

The study population was divided into two cohorts based on whether individuals were dispensed antibiotics (ATC-codes beginning with J01) within 3 days before to 7 days after the index date (AB+OCS cohort) or not (OCS-only cohort). The range of 3 days before until 7 days after the index date was chosen because medication prescribed simultaneously may be dispensed at different times due to factors such as pharmacy stock. Additionally, physicians might wait to observe initial persistence or worsening of symptoms before prescribing an antibiotic.

The primary outcome was treatment failure, defined as a new dispense of prednisone, prednisolone or antibiotics between 15 and 30 days after the index date, as these are the medications typically prescribed when initial treatment is unsuccessful. Since the dispensing of OCS and antibiotics in such cases may reflect different clinical scenarios, results will also be reported separately for treatment failure involving a new OCS dispense and those involving a new antibiotic dispense. In addition, the separate reporting of treatment failure based on new OCS dispenses will serve as sensitivity analysis to rule out any potential association between antibiotic dispensing at the index and the likelihood of subsequent antibiotic dispense.

As a secondary outcome, time until a second exacerbation is defined as another dispense of prednisone or prednisolone 31–365 days after the index date. This study setup was inspired by the work of Wang *et al*, who conducted a real-world cohort study on the effects of antibiotic treatment for acute COPD exacerbations in outpatients.^11^

### Covariates

Asthma treatment steps were defined following the adult asthma guidelines from the Dutch College of General Practitioners (NHG)^25^ and the GINA recommendations^26^:

- Step 1: SABA or low-dose ICS plus formoterol.
- Step 2: Low-dose ICS without LABA or LAMA.
- Step 3: Medium dose ICS, low dose ICS plus LABA or LAMA, or montelukast in combination with inhaled asthma medication from a lower step.
- Step 4: High dose ICS or medium to high dose ICS plus LABA.
- Step 5: Triple therapy (ICS plus LABA plus LAMA) or biologics.
- Step 0: No current prescriptions meeting any of the criteria of treatment steps 1–5.

Individuals were classified into these treatment steps based on medication dispensed the year before the index date. The most recent dispense was prioritised in identifying ICS dosage.

Further covariates included in the analyses were age, sex (male or female—as specified in the national Personal Records Database), season (March–May (spring; hay fever peak season); June–August (summer); September–November (fall); December–February (winter; influenza peak season)), index year, number of SABA dispenses in the year before index date (as proxy for asthma control), number of ICS dispenses (including ICS plus LABA) in the year before index date as interaction with treatment step (as proxy for adherence), and presence of arthritis, atopic disease, cardiovascular disease, diabetes, mental health problems, peptic ulcer and gastro-oesophageal reflux disease and thyroid disease (defined by ≥2 dispenses of medication used to treat the comorbidity within the year before the index date; see online supplement 1).

### Statistical methods

Descriptive statistics (eg, mean/median and SD/IQR) were used to compare baseline characteristics. Inverse probability of treatment weighting (IPTW), using logistic regression to estimate propensity scores, was applied to adjust for potential covariate imbalances between the cohorts.^27^ In addition, the weighted logistic and Cox proportional hazard regression models also adjusted for covariates and interaction terms (online supplement 2), making them doubly robust. 95% CIs were obtained by bootstrapping (1000 iterations).^28^

To explore the robustness of our findings across various subgroups, we conducted stratified analyses by type of antibiotic, age, sex, treatment steps, year and season at baseline. Moreover, to identify whether these baseline characteristics modified the association between antibiotic exposure and the outcomes, interaction terms with overall antibiotic, macrolide, penicillin and tetracycline exposure were added one-by-one to the adjusted (unweighted) regression models. Additional analyses, assessing the potential effect of unmeasured confounders, can be found in online supplement 3.

## Results

### Study population selection

**Figure 2:**
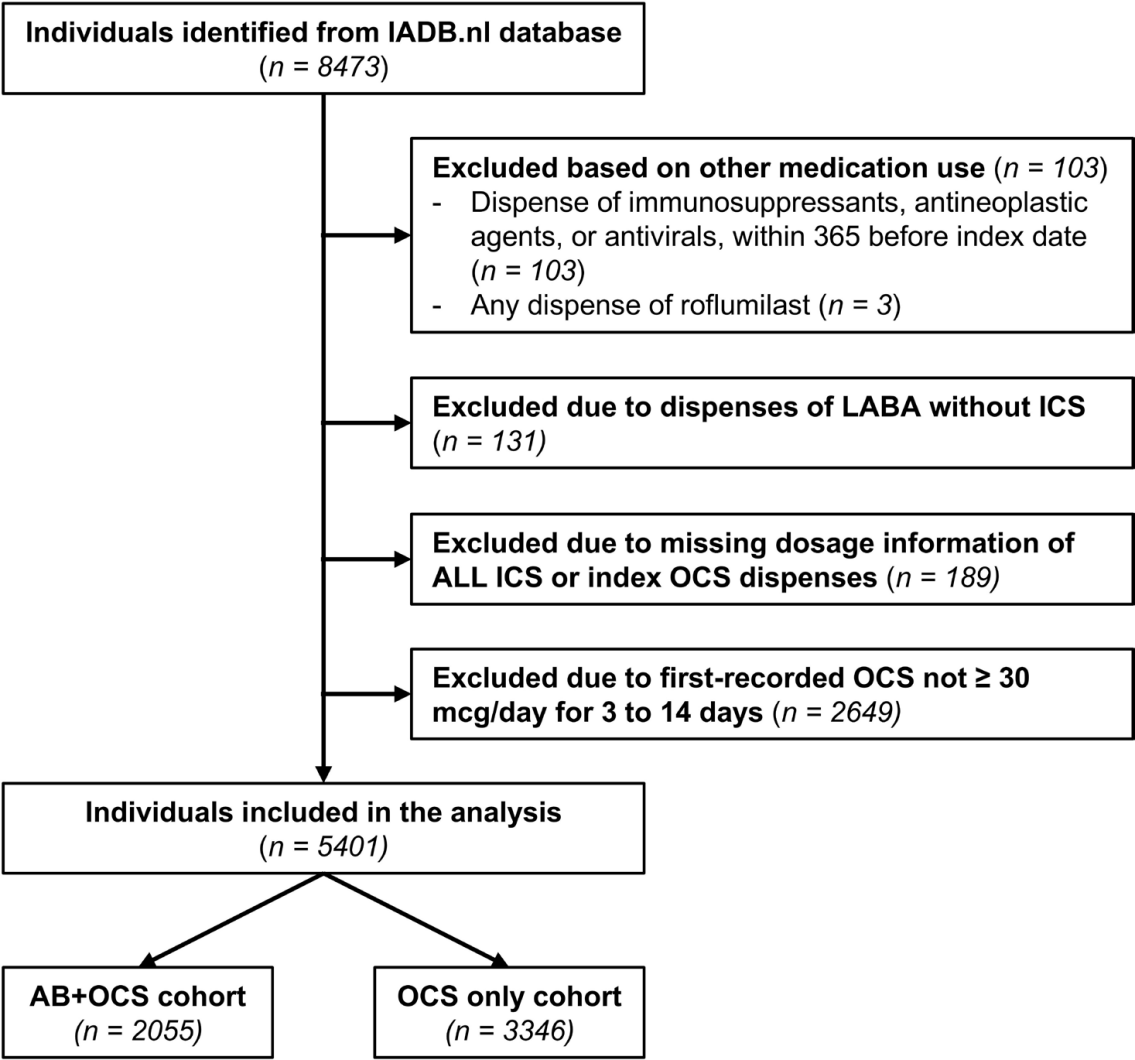
Patient selection flow chart. AB, antibiotic; ICS, inhaled corticosteroid; LABA, long-acting selective β2-adrenoreceptor agonists; OCS, oral corticosteroid.

Initially, 8473 individuals were identified from the IADB.nl community pharmacy dispensing database. We excluded 398 individuals based on dispenses of immunosuppressants, antineoplastic agents, antivirals or roflumilast (n=103), use of LABA without ICS (n=131) or missing data on all ICS or index OCS dispenses (n=189), and 2649 were excluded due to their first-recorded OCS dispense being <30 mg/day or not for 3–14 days (figure 2).

### Baseline characteristics

**Figure 3:**
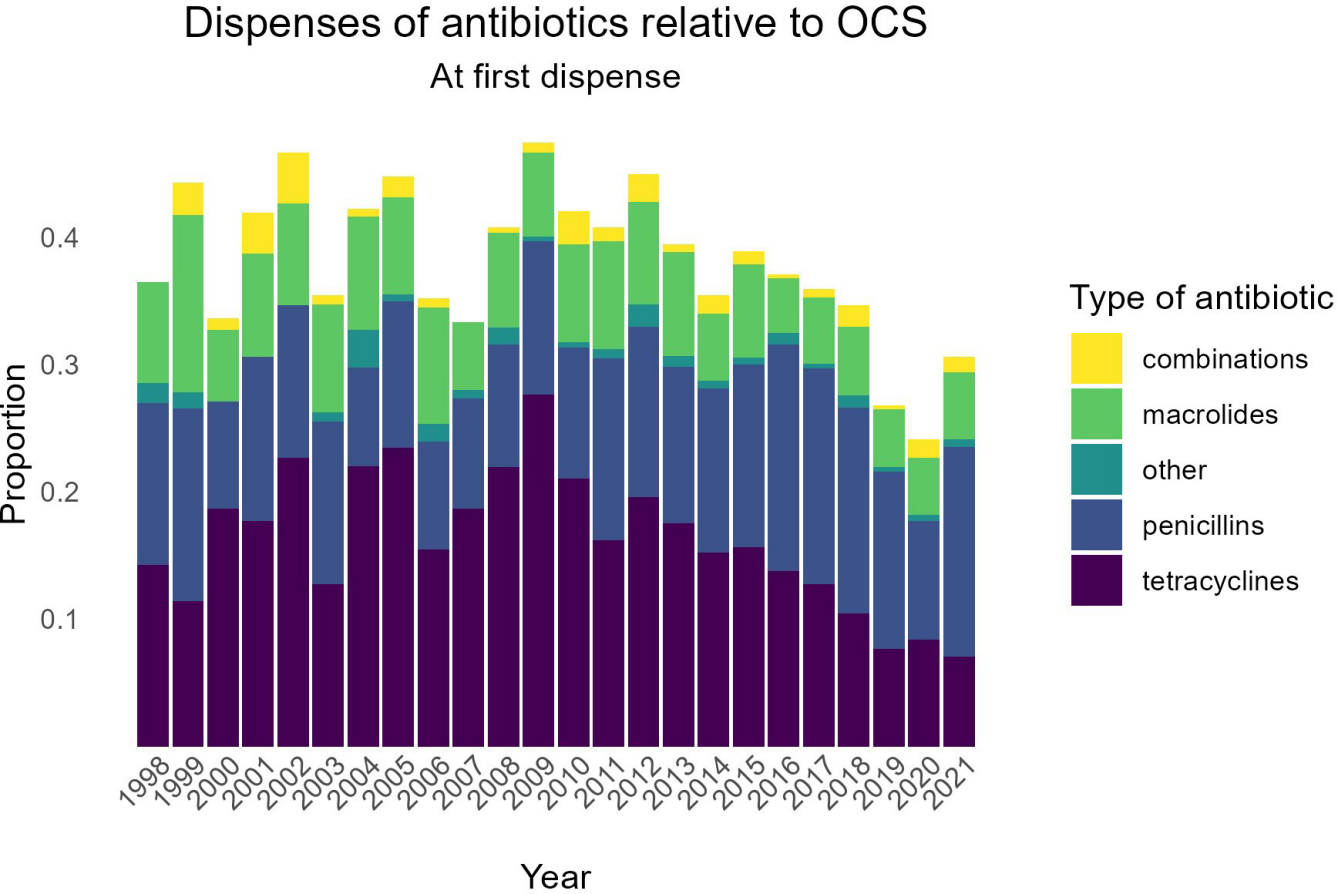
Dispenses of antibiotics at baseline relative to OCS per year. OCS, oral corticosteroid.

From the 5401 included individuals, 2055 (38%) received antibiotics (AB+OCS cohort) and the remaining 3346 (62%) were enrolled in the OCS-only cohort. In 2009, the proportion of first exacerbations for which antibiotics were dispensed (thus relative to first OCS dispense) was at its highest (47%). Since then, there seems to be a decreasing trend, with the lowest proportion (24%) occurring in 2020 (figure 3). Tetracyclines (40%) were the most commonly dispensed antibiotic in this study, followed by penicillins (35%) and macrolides (20%) (online supplement 4).

In total, 2035 participants were male. The mean (SD) age was 32.7 (8.5) years in the OCS-only cohort and 33.6 (8.6) years in the AB+OCS group. We identified no individuals who met the inclusion criteria before 1998, mainly due to missing OCS dosage information or dosages lower than 30 mg per day. Demographic and treatment characteristics were similar between the two cohorts (table 1). After IPTW, the standardised mean differences between the cohorts were <0.003 for all covariates (online supplement 5).

**Table 1:** Patient and treatment characteristics at baseline

	OCS-only cohort		AB+OCS cohort	
	(n=3346)		(n=2055)	
	n	%	n	%
Sex				
Male	1252	37	783	38
Age				
16–25	820	25	445	22
26–35	1045	31	584	28
36–45	1481	44	1026	50
Asthma treatment step				
1	787	24	483	24
2–3	1037	31	593	29
4–5	1522	45	979	48
Antibiotic treatment during the first exacerbation*				
Macrolides	–		431	20
Penicillins	–		748	36
Tetracyclines	–		918	45
Other	–		63	3
Comorbidities				
Arthritis	365	11	279	14
Atopic disease	1483	44	857	42
Cardiovascular disease	246	7	170	8
Diabetes	70	2	60	3
Gastro-oesophageal reflux disease and peptic ulcer	436	13	290	14
Mental health problems	508	15	382	19
Thyroid disease	75	2	58	3
Year of index date†				
1998–2005	520	16	358	17
2006–2013	1295	39	915	45
2014–2021	1531	46	782	38
Season				
Spring	823	25	471	23
Summer	690	21	373	18
Fall	892	27	506	25
Winter	941	28	705	34

* The specific antibiotics and corresponding frequencies can be found in online supplement 4.

† The study period ranges from 1994 to 2021, but no individuals in the IADB.nl database met the inclusion criteria before 1998.

AB, antibiotic; OCS, oral corticosteroid.

### Treatment failure

Any treatment failure (ie, receiving a new dispense of OCS or antibiotics 15–30 days after the index date) was experienced by 198 participants (5.9%) in the OCS-only and 130 (6.3%) in the AB+OCS cohort. The crude OR of failing treatment when being dispensed antibiotics vs no antibiotics was 1.07 (95% CI 0.85 to 1.35). The doubly robust estimated OR was 1.36 (95% CI 0.81 to 2.16).

Treatment failure with a new OCS dispense occurred in 70 (2.1%) participants of the OCS-only cohort and 58 (2.8%) of the AB+OCS cohort. The crude OR of treatment failure with new OCS was 1.36 (95% CI 0.96 to 1.93) in the AB+OCS cohort relative to the OCS-only cohort, and the doubly robust OR was 3.68 (95% CI 1.71 to 9.55).

Lastly, treatment failure with a new AB dispense occurred in 152 (4.5%) participants of the OCS-only cohort and 106 (5.2%) of the AB+OCS cohort. The crude OR of treatment failure with new AB was 1.14 (95% CI 0.89 to 1.47), and the doubly robust OR was 1.11 (95% CI 0.61 to 1.87).

### Risk of second exacerbation

A second exacerbation, recorded as being dispensed OCS between 31 and 365 days after the index, occurred among 682 participants in the OCS-only cohort (20.4%) and 488 participants in the AB+OCS cohort (23.7%). The crude HR for having a second exacerbation in the AB+OCS cohort versus the OCS-only cohort was 1.19 (95% CI 1.06 to 1.34). The doubly robust HR was 1.20 (95% CI 0.92 to 1.56).

### Stratified analyses

**Figure 4:**
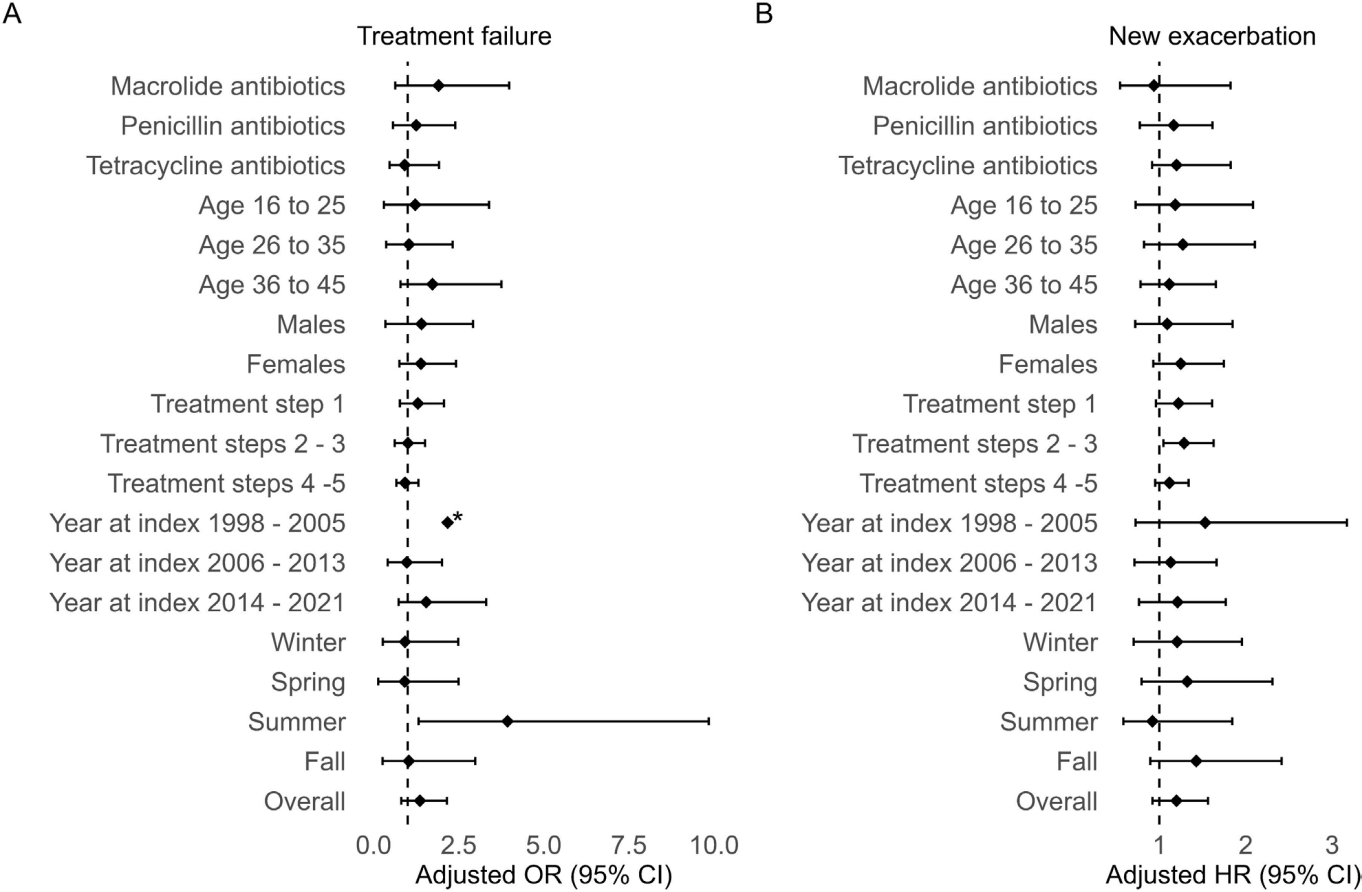
Forest plot of (**A**) the weighted adjusted OR of asthma exacerbation treatment failure and (**B**) the weighted adjusted HR of having a second exacerbation, when being dispensed a short course of oral corticosteroids plus antibiotics versus not being dispensed antibiotics, within subgroups. *No CI is displayed for treatment failure in the ‘Year at index 1988–2005’ subgroup, as it was too wide to be displayed in this figure.

Sensitivity analyses determined the effect within subgroups based on the type of antibiotic, age, sex, treatment step, year and season, while adjusting for potential confounders. The only significant effect of antibiotics on treatment failure was found for the subgroup having their first exacerbation in summer (OR 3.93, 95% CI 1.32 to 9.85). For having a second exacerbation, the only significant effect was found in the subgroups with maintenance asthma treatment in steps 2–3 (HR 1.29, 95% CI 1.05 to 1.63). Both of these significant effects are in favour of not dispensing antibiotics (figure 4). No significant interaction effects were present between these subgroups and the dispensed antibiotics (online supplemental Table S6.1 and S6.2).

## Discussion

In this study, we evaluated the real-world added value of antibiotics alongside OCSs in treating acute asthma exacerbations in adults, taking into account potential confounding. Overall, we found no beneficial effect of using antibiotics in addition to OCSs for acute asthma exacerbations on treatment failure within 2–4 weeks or on developing a second exacerbation within a year, and a negative effect regarding new prescriptions of OCSs within 2–4 weeks. The results remained robust across various subgroups and for potential unmeasured confounding.

The results are in line with comparable studies, especially those conducted in the primary care setting. Murray *et al* found a slightly decreased risk of having a primary care consultation for asthma or wheeze within 2 weeks after penicillins, but not after macrolides, and an increase in exacerbations among adults within 6 weeks after macrolides or penicillins.^10^ In the hospital setting, several clinical trials have been conducted to assess the added value of antibiotics. The AMAZES trial reported some benefits of long-term preventative macrolides in the management of asthma.^29^ A trial by Johnston *et al* on the treatment of exacerbations with a macrolide (telithromycin) compared with placebo reported improvement of asthma symptoms, but not of peak expiratory flow, and even found an increase in nausea.^29 30^ In contrast, the AZALEA trial found no benefit of azithromycin (a macrolide) versus placebo on lung function, symptom score or quality of life, for treatment of acute asthma exacerbations.^31^ In addition, the cohort study of Stefan *et al* even reported a small increase in hospital stay duration and costs after antibiotic treatment.^15 16 31^

Our study found that antibiotics were dispensed for 38% of first recorded asthma exacerbations, which is only 10% lower than for COPD exacerbations.^11^ That is surprisingly high, given that routine treatment with antibiotics for asthma exacerbations is not supported by the Dutch guidelines.^25^ Yet these numbers are in line with prescription rates found by other researchers.^10^ We cannot rule out that some of the dispensed antibiotics may be justified by other causes, simultaneously presenting with, but unrelated to, the asthma exacerbation. Even though such an unrelated additional dispense of antibiotics means asthma control could be negatively affected by another cause, the unrelated antibiotics dispense could still decrease the likelihood of treatment failure or a new exacerbation (if such a beneficial effect exists). In addition, figure 3 shows a decreasing trend in antibiotics prescribed next to OCS for acute asthma exacerbations from 47% in 2009 to 24% in 2020, supporting the idea that a substantial proportion of antibiotic prescriptions might have been redundant. More importantly, the long-term global health effects of overprescribing antibiotics are serious, as they can lead to the development of drug-resistant pathogens. According to the WHO, antimicrobial resistance is one of the top public health and development threats and has been directly responsible for more than 1.2 million deaths in 2019 alone.^32^

Although the point estimates in our study suggest no beneficial effect of antibiotics next to OCSs on asthma exacerbations, the lower bounds of 95% CIs are mostly below one, indicating that we cannot with certainty rule out the possibility of a minor beneficial effect. Another limitation in this study, like in all observational studies, is that residual confounding cannot be ruled out. Especially confounding by indication forms a challenge in this study, as patients with lesser controlled asthma, more severe exacerbations or signs of bacterial infection (eg, purulent sputum, fever, or increased C-reactive protein (CRP) in blood) could be more likely to both be prescribed antibiotics and have worse outcomes. Online supplement 3 shows that the analyses were quite robust in case of potential unmeasured confounding (of a single binary confounder with a 10% prevalence difference), enhancing the reliability of the findings. In addition, the findings of this study showed robustness across various subgroups. Another limitation of this study is the lack of data on hospitalisations and medication administered within hospital settings. As a result, the most severe asthma exacerbations might be missed. However, in the Netherlands, asthma is predominantly treated by general physicians, so it likely will entail only a minor proportion. Lastly, the inclusion of participants aged 16–45 limits the direct generalisability of our findings to younger and older populations with asthma. Nonetheless, the substantial extent of overprescription observed in this study suggests that this issue likely extends to wider age ranges. Of note, it has been reported that bacterial lung infections are relatively more common among young children and older patients with chronic lung disease, while Murray *et al* reported the odds of being prescribed antibiotics for asthma exacerbations to be lower in children and increased in the elderly.^10 33^ In short, our results may, therefore, not be applicable to asthma patients outside the studied age range, which should be taken into account when generalising these results and warrants further research.

This study benefited from a large, heterogeneous study population, derived from the IADB.nl database, which has been proven representative of the Dutch population and is virtually complete, except for hospitalisations.^19 20^ Although one might argue that dispenses of prednisolone/prednisone serving as proxy for exacerbations could result in misclassification, it is in line with the definition of asthma exacerbations as has been proposed by a group of experts: ‘a worsening of asthma requiring the use of systemic corticosteroids to prevent a serious outcome’.^34^ Additionally, similar research on COPD exacerbations was able to show a beneficial association between doxycycline dispensing and treatment failure in a considerably smaller study population (n=1105).^11^ This strengthens our belief that if a clinically relevant benefit of antibiotics on acute asthma exacerbations regarding treatment failure or following exacerbations had existed, our study would have been able to demonstrate a relationship.

For further research, it would be interesting to find out what drives physicians to prescribe antibiotics for exacerbations of asthma, and assess which of those factors are predictive of beneficial short-term and long-term treatment outcomes. Moreover, a cost-effectiveness analysis following treatment failure might reveal extra costs associated with antibiotic use in this context. Such findings could provide physicians, hospitals and policymakers with explicit evidence of the financial burden linked to antibiotic prescriptions for asthma exacerbations, offering additional motivation to avoid unnecessary use. Next, a previous systematic review found that bacteria were detected in only 7% of asthma exacerbations, while we found that 38% of cases were prescribed antibiotics.^9^ Therefore, linkage with, for example, medical records could give insight into whether prescriptions of antibiotics are justified by bacterial infection (eg, by CRP-test results or incorporating variables such as fever and sputum colour), thus helping to rule out confounding by indication (at least partially) and providing further insight into the effectiveness of antibiotic prescription against guideline recommendations. Lastly, it would be interesting to study antibiotic effectiveness for different subtypes of asthma, as differences in biological mechanisms might explain heterogeneity in treatment effectiveness. Nevertheless, taking into account the evidence currently available and the threats of antimicrobial resistance, we recommend against the use of antibiotics for managing asthma exacerbations, unless presented with clear signs of bacterial infection.

In conclusion, this study found no beneficial effect from the addition of antibiotics to OCS treatment for managing acute asthma exacerbations in adults in Dutch primary care. Usage of antibiotics did not reduce the risk of treatment failure or the time until the next exacerbation. Taking into account available literature, we suggest that antibiotics should not be used for treating acute asthma exacerbations in primary care among adults, unless there are clear signs of bacterial infection.

## Supplementary material

10.1136/bmjresp-2025-003506 online supplemental file 1

## Data Availability

Data may be obtained from a third party and are not publicly available.
