# Supplementary material for: Real-world effectiveness of antibiotics in addition to oral corticosteroids for managing asthma exacerbations in adults
Source: BMJ Open Respir Res. 2025 Oct 20;12(1):e003506. doi: 10.1136/bmjresp-2025-003506 (PMC12542710; doi:10.1136/bmjresp-2025-003506)
Supplement: online supplemental file 1 [file bmjresp-12-1-s001.docx]

**Supplementary material**

#

# Overview:

Supplement 1: ATC-codes per comorbidity

Supplement 2: Model specifications

Supplement 3: Potential effects of unmeasured confounding

# Supplement 4: Frequencies of antibiotics at baseline & after treatment failure

# Supplement 5: Standardised mean difference plot

Supplement 6: Interaction analyses

# Supplement 1 - ATC codes per comorbidity

# Table S1: ATC-codes per comorbidity

| ATC-codes per condition | |
| --- | --- |
| Drugs used for… | **ATC-codes** |
| Arthritis | M01 or M02 |
| Atopic diseases^1^ | D07, D11AH, R01AC, R01AD, R06, S01GX |
| Cardiovascular disease | C01AA05, C01DA, C02, C03, C07, C08, C09 |
| Diabetes | A10 |
| Mental health problems^2^ | N05B, N05C, N06A, N06D |
| Peptic ulcer and GERD | A02B |
| Thyroid disease | H03 |

Abbreviations: ATC-codes = Anatomic Therapeutic Chemical -codes; GERD = gastroesophageal reflux disease.

^1^Includes allergies, allergic rhinitis, allergic conjunctivitis, and dermatitis

^2^Includes depression, anxiety, dementia, and insomnia

# Supplement 2 – Model specifications

**Exposure model**

exposure ~ trstep_cat + ns(age_at_index, df=3) + sex + arthritis + ATD + CVD + diabetes + GERD + MHP + thyroid + season + nprescics*trstep_cat + nprescsaba + ns(indexyear, df=3)

**Outcome model for treatment failure**

trfailure ~ exposure + trstep_cat*exposure + trstep_cat + ns(age_at_index, df=3) + sex + arthritis + ATD + CVD + diabetes + GERD + MHP + thyroid + season + nprescics*trstep_cat + nprescsaba + ns(indexyear, df=3)

**Outcome model for time until second exacerbation**

Surv(scexc_t, scexc) ~ exposure + trstep_cat*exposure + trstep_cat + ns(age_at_index, df=3) + sex + arthritis + ATD + CVD + diabetes + MHP + GERD + thyroid + season + nprescics*trstep_cat + nprescsaba + ns(indexyear, df=3)

# Abbreviations: trstep_cat = treatment step category (1; 2 – 3; 4 - 5); ATD = atopic disease; CVD = cardiovascular disease; GERD = gastroesophageal reflux disease & peptic ulcer; MHP = mental health problems; thyroid = thyroid disease; nprescics = number of ICS dispenses (including ICS plus LABA); nprescsaba = number of SABA dispenses; ns = natural spline; df = degrees of freedom.Supplement 3 – Potential effects of unmeasured confounding

The R package ‘tipr’ was used to assess robustness against unmeasured confounding. We considered confounder-outcome relationships with ORs/HRs ranging from 1 to 10. As example, tobacco smoking status was used, for which prevalences in exposed (antibiotic) and non-exposed (OCS only) cohorts were obtained from a study by Murray et al. (2021, Ref. 10 of manuscript) conducted between 2004 and 2014 in the UK. In addition, we investigated the effect of a 10% difference in prevalence, using 20 vs. 30%, and 45 vs. 55%.

Of note: these results are not limited to smoking but can be interpreted as any binary confounder. This analysis relies on the assumption that the unmeasured confounder is independent of the included covariates. However, were this assumption violated, the effect of the unmeasured confounder would most likely overestimated, due to relatedness with confounders already adjusted for (Groenwold RH, Hoes AW, Nichol KL, Hak E. Quantifying the potential role of unmeasured confounders: the example of influenza vaccination. Int J Epidemiol. 2008;37(6):1422-9.9).


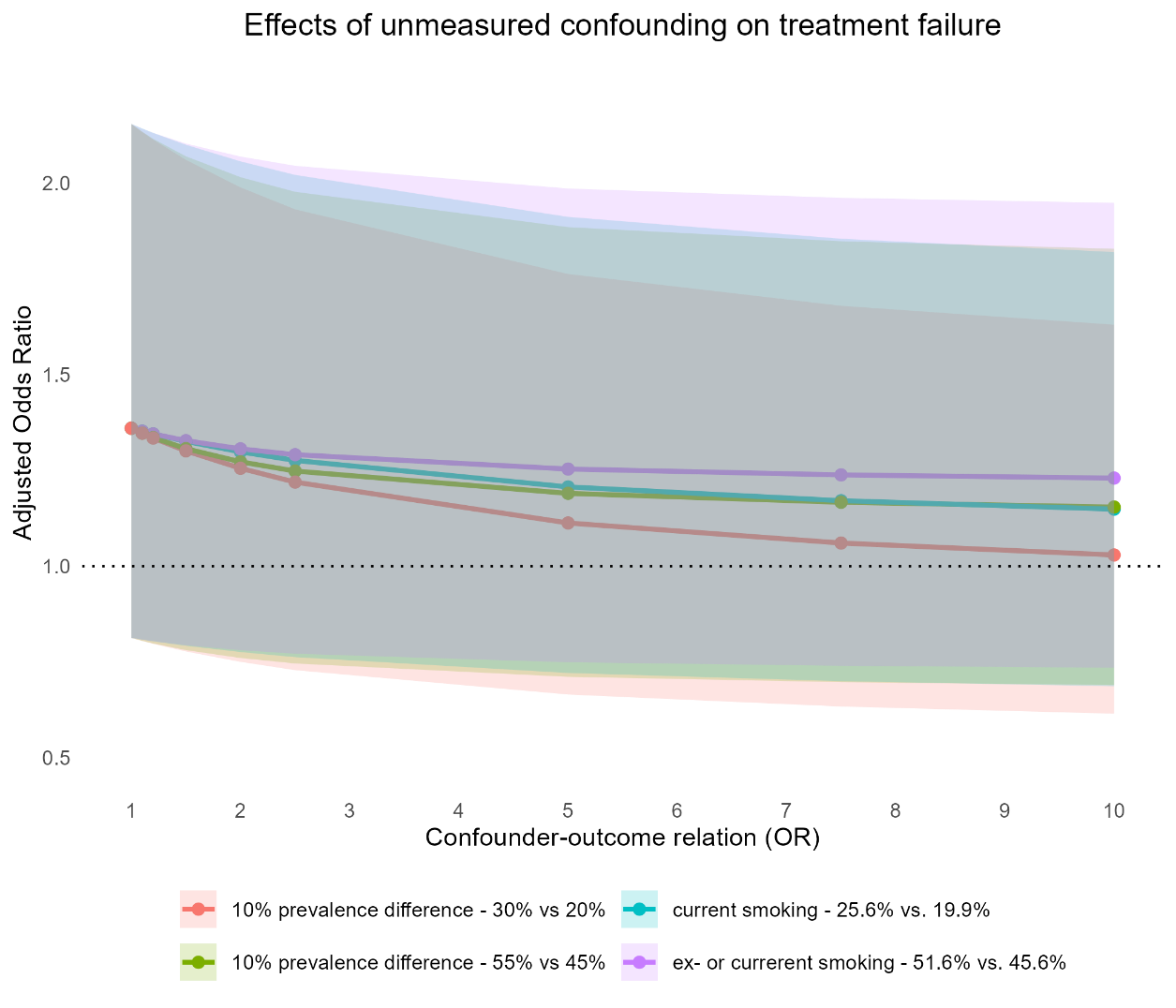
**Figure S3A:** Effect of AB next to OCS on treatment failure, under various hypothetical scenarios of unmeasured (binary) confounding, represented with different prevalences between the OCS only vs. AB+OCS cohorts.

**Figure S3B:** Effect of AB next to OCS on second exacerbations, under various hypothetical scenarios of unmeasured (binary) confounding, represented with different prevalences between the OCS only vs. AB+OCS cohorts.


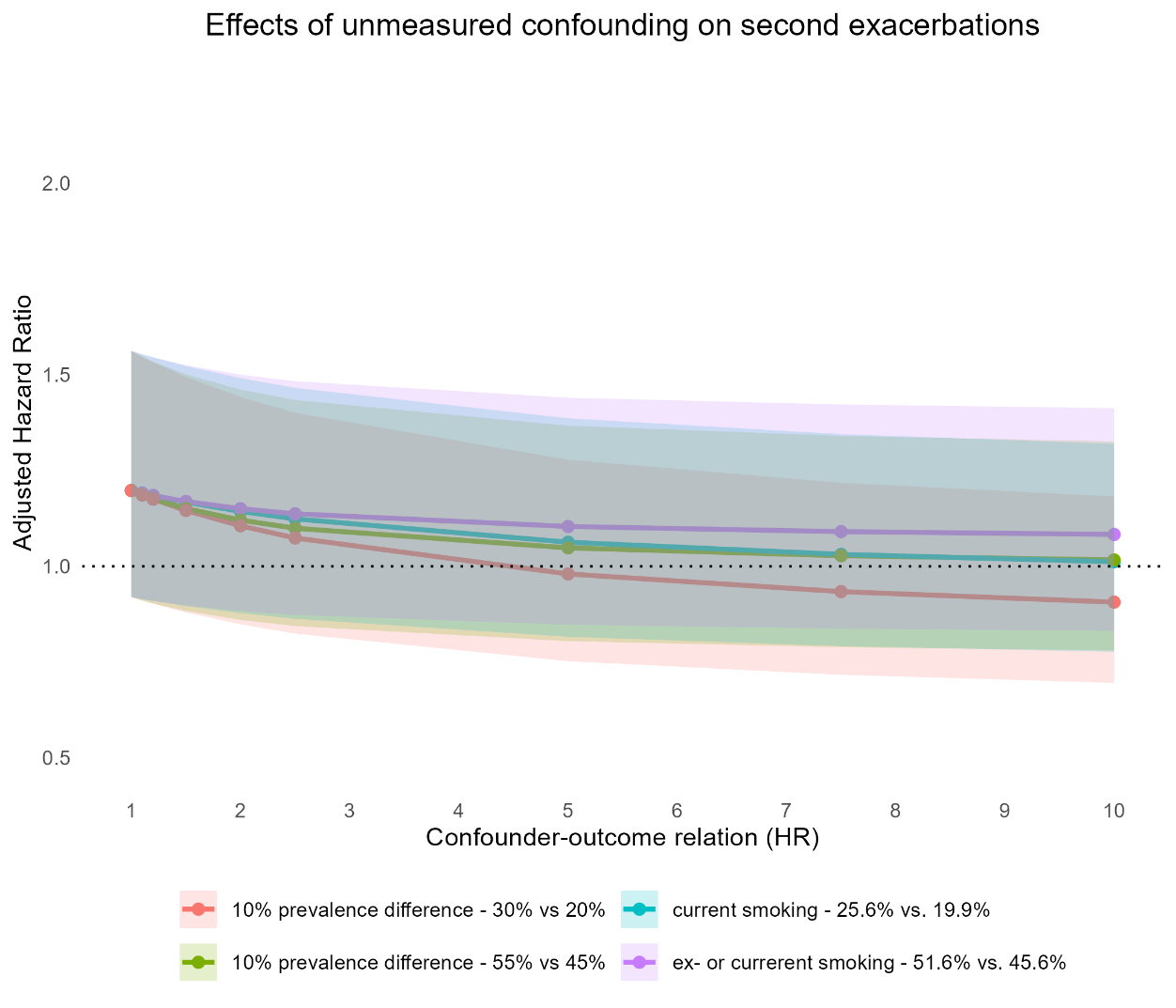


These analyses show that if a confounder with prevalences like current tobacco smoking increased the odds of treatment failure and hazard of having a second exacerbation by 10 times, the effect of being dispensed antibiotics on treatment failure (aOR (95% CI): 1.15 (0.67 – 1.84)) and having a second exacerbation (aHR (95% CI): 1.01 (0.77 – 1.31)) would both remain insignificant. This stays true when including former tobacco smoking. Even with a 10% prevalence difference and a confounder-outcome association (OR/HR) of 10, the results would remain insignificant for treatment failure and hazard of a second exacerbation.

# Supplement 4 - Frequencies of antibiotics at baseline & after treatment failure

**Table S4:** The number of dispenses at baseline and in the 15 – 30 days after index date (indicates treatment failure) per antibiotic

| Type of antibiotic | ATC-code | Drug name | Dispenses at baseline ^1^  *n = 2181*  *(n, % >=1)* | Dispenses for treatment failure ^2^  *n = 281*  *(n, % >=1)* |
| --- | --- | --- | --- | --- |
| Tetracyclines | J01AA02 | Doxycycline | 928 (43) | 55 (20) |
|  | J01AA08 | Minocycline | 1 | - |
| Penicillins | J01CA04 | Amoxicillin | 494 (23) | 52 (19) |
|  | J01CE02 | Phenoxymethylpenicillin | 1 | 1 |
|  | J01CE05 | Pheneticillin | 3 | 5 (2) |
|  | J01CF05 | Flucloxacillin | 10 | 4 (1) |
|  | J01CR02 | Amoxicillin and beta-lactamase inhibitor | 257 (12) | 47 (17) |
| Trimethoprims | J01EA01 | Trimethoprim | 7 | 4 (1) |
|  | J01EE01 | Sulfamethoxazole and trimethoprim | 21 (1) | 6 (6) |
| Macrolides | J01FA01 | Erythromycin | 3 | 1 |
|  | J01FA06 | Roxithromycin | 6 | - |
|  | J01FA09 | Clarithromycin | 221 (10) | 35 (12) |
|  | J01FA10 | Azithromycin | 191 (9) | 35 (12) |
| Lincosamides | J01FF01 | Clindamycin | 3 | 4 (1) |
| Fluoroquinolones | J01MA01 | Ofloxacin | 3 | - |
|  | J01MA02 | Ciprofloxacin | 14 (1) | 5 (2) |
|  | J01MA06 | Norfloxacin | 1 | 2 (1) |
|  | J01MA12 | Levofloxacin | 3 | 1 |
|  | J01MA14 | Moxifloxacin | 4 | 1 |
| Other antibacterials | J01XD01 | Metronidazole | 1 | - |
|  | J01XE01 | Nitrofurantoin | 9 | 21 (7) |
|  | J01XX01 | Fosfomycin | - | 2 (1) |

^1^ Dispensed within 3 days before till 7 days after index date

^2^ Dispensed within 15 to 30 days after index date

# Supplement 5 – Standardised mean difference plot


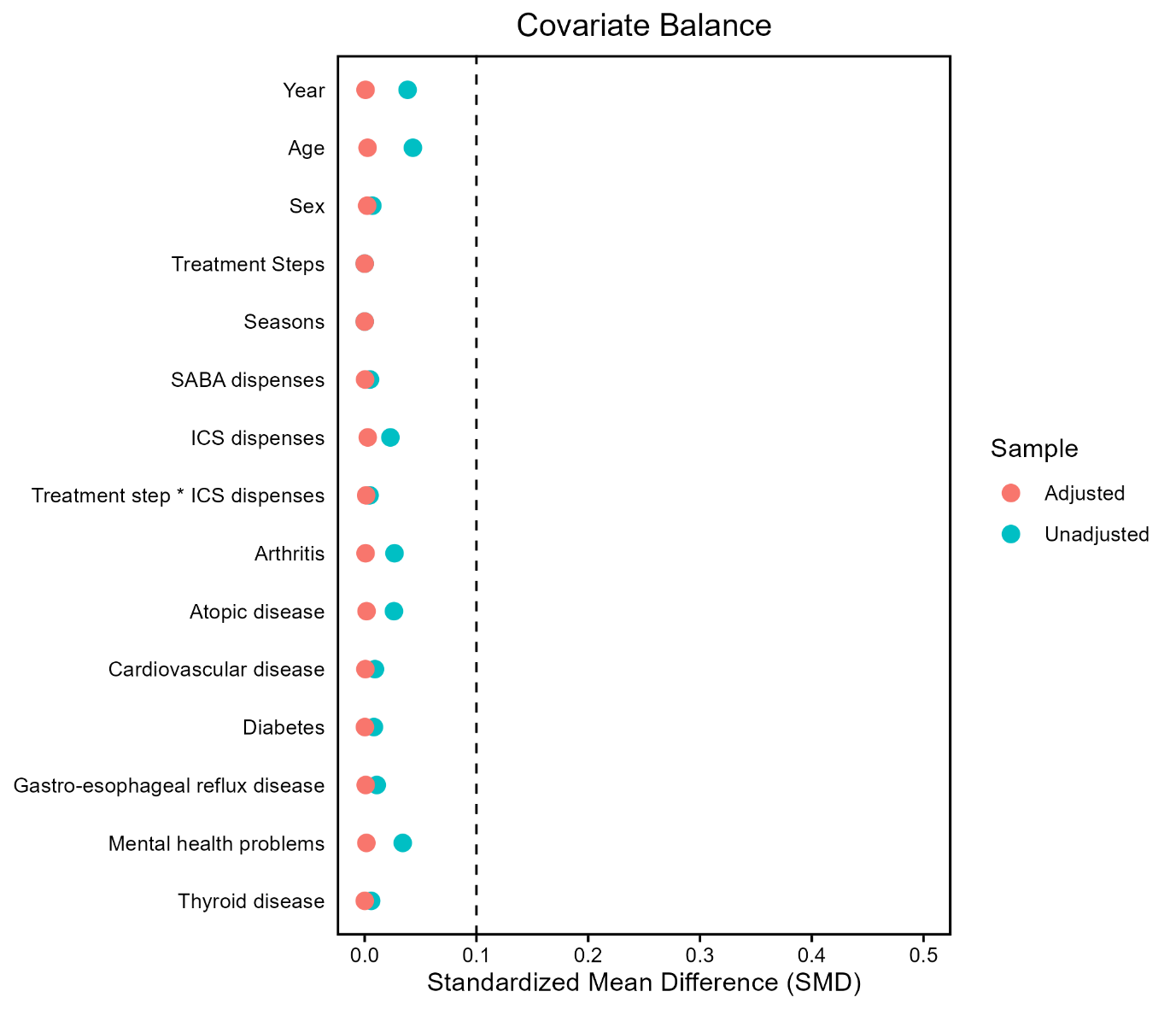


# Supplement 6 – Interaction analyses

|  | Any antibiotic | Macrolide | Penicillin | Tetracycline |  |
| --- | --- | --- | --- | --- | --- |
|  | *p-value* | *p-value* | *p-value* | *p-value* |  |
| Age |  |  |  |  |  |
| At index (continuous) | 0.59 | 0.56 | 0.11 | 0.20 |  |
| Sex |  |  |  |  |  |
| Female | Ref. | Ref. | Ref. | Ref. |  |
| Male | 0.11 | 0.43 | 0.79 | 0.27 |  |
| Year |  |  |  |  |  |
| At index (continuous) | 0.47 | 0.41 | 0.47 | 0.80 |  |
| Season |  |  |  |  |  |
| Winter (Dec – Feb) | Ref. | Ref. | Ref. | Ref. |  |
| Spring (Mar – May) | 0.42 | 0.93 | 0.79 | 0.72 |  |
| Summer (Jun – Aug) | 0.40 | 0.61 | 0.89 | 0.52 |  |
| Fall (Sep – Nov) | 0.73 | 0.52 | 0.21 | 0.31 |  |
| Asthma treatment step |  |  |  |  |  |
| 1 | Ref. | Ref. | Ref. | Ref. |  |
| 2 – 3 | 0.47 | 0.18 | 0.27 | 0.13 |  |
| 4 – 5 | 0.33 | 0.42 | 0.96 | 0.28 |  |

**Table S6.1:** Interactions separately added to the unweighted adjusted regression analysis for treatment failure

Abbreviations: Ref. = Reference

|  | Any antibiotic | Macrolide | Penicillin | Tetracycline |  |
| --- | --- | --- | --- | --- | --- |
|  | *p-value* | *p-value* | *p-value* | *p-value* |  |
| Age |  |  |  |  |  |
| At index (continuous) | 0.66 | 0.57 | 0.34 | 0.78 |  |
| Sex |  |  |  |  |  |
| Female | Ref. | Ref. | Ref. | Ref. |  |
| Male | 0.67 | 0.55 | 0.57 | 0.92 |  |
| Year |  |  |  |  |  |
| At index (continuous) | 0.74 | 0.41 | 0.41 | 0.42 |  |
| Season |  |  |  |  |  |
| Winter (Dec – Feb) | Ref. | Ref. | Ref. | Ref. |  |
| Spring (Mar – May) | 0.67 | 0.76 | 0.58 | 0.76 |  |
| Summer (Jun – Aug) | 0.94 | 0.28 | 0.78 | 0.28 |  |
| Fall (Sep – Nov) | 1.00 | 0.69 | 0.58 | 0.69 |  |
| Asthma treatment step |  |  |  |  |  |
| 1 | Ref. | Ref. | Ref. | Ref. |  |
| 2 – 3 | 0.81 | 0.63 | 0.73 | 0.63 |  |
| 4 – 5 | 0.47 | 0.22 | 0.63 | 0.22 |  |

**Table S6.2:** Interactions separately added to the unweighted adjusted regression analysis for second exacerbation

Abbreviations: Ref. = Reference
